# Health promotion services for patients having non-comminicable diseases: Feedback from patients and health care providers in Cape Town, South Africa

**DOI:** 10.1186/1471-2458-12-503

**Published:** 2012-07-04

**Authors:** Whadi-ah Parker, Nelia P Steyn, Naomi S Levitt, Carl J Lombard

**Affiliations:** 1 Centre for the Study of the Social and Environmental Determinants of Nutrition, Population Health, Health Systems and Innovation, Human Sciences Research Council, Private Bag X9182, Cape Town, 8000, South Africa; 2 Diabetes and Endocrine Unit, Department of Medicine, University of Cape Town, Obervatory, 7925, South Africa; 3 Biostatistics Unit, South African Medical Research Council, PO Box 19070, Tygerberg, 7505, Cape Town, South Africa

**Keywords:** Patient preferences, Health education materials, Health education methods, Chronic diseases of lifestyle, Lifestyle modification

## Abstract

**Background:**

Due to a paucity of data regarding the availability and efficacy of equipment, health promotion methods and materials currently used by health professionals for the management of patients with non-communicable diseases (NCDs) at primary health care (PHC) facilities in Cape Town, an audit was undertaken.

**Methods:**

A multi-centre cross-sectional study was undertaken to interview patients (n = 580) with NCDs at 30 PHC facilities. A questionnaire was used to obtain information on preferences for health promotion methods for lifestyle modification. Individual semi-structured interviews were conducted with selected health professionals (n = 14) and captured using a digital recorder. Data were transferred to the Atlas ti software programme and analysed using a thematic content analysis approach.

**Results:**

Blood pressure measurement (97.6%) was the most common diagnostic test used, followed by weight measurement (88.3%), urine (85.7%) and blood glucose testing (80.9%). Individual lifestyle modification counselling was the preferred health education method of choice for the majority of patients. Of the 64% of patients that selected chronic clubs/support groups as a method of choice, only a third rated this as their first choice. Pamphlets, posters and workshops/group counselling sessions were the least preferred methods with only 9%, 13% and 11% of patients choosing these as their first choice, respectively. In an individual counselling setting 44.7% of patients reported that they would prefer to be counselled by a doctor, followed by a nurse (16.9%), health educator (8.8%) and nutrition advisor (4.8%). Health professionals identified numerous barriers to education and counselling. These can be summarised as a lack of resources, including time, space and equipment; staff-related barriers such as staff shortage and staff turnover; and patient-related barriers such as patient load and patient non-compliance.

**Conclusion:**

The majority of patients attending PHC facilities want to receive lifestyle modification education. There is not however, one specific method that can be regarded as the gold standard. Patients’ preferences regarding health education methods differ, and they are more likely to be susceptible to methods that do not involve much reading. Health education materials such as posters, pamphlets and booklets should be used to supplement information received during counselling or support group sessions.

## Background

As in many other developing countries, the prevalence of chronic NCDs is increasing in South Africa [1]. Non-communicable diseases (NCDs) account for the largest proportion of adult mortality in the Western Cape Province of which Cape Town is the major urban centre [2]. Approximately 23 395 hypertensive and 13 338 diabetic patients attend primary health care (PHC) facilities in Cape Town every month [2]. These facilities should therefore be equipped to provide information, education and counselling on lifestyle modification to patients. PHC facilities should also have adequate access to equipment and health promotion materials needed for patients with NCDs or related risk factors.

In 2003, Reagon *et al.* conducted a national survey of PHC facilities in South Africa [3]. This included the availability of equipment and health promotion materials which they referred to as Information, Education and Communication (IEC) materials. PHC facilities in the Western Cape appeared to be in an advantageous position since at least one of each of the equipment that deals with diagnoses of NCDs (adult scale; stethoscope; baumanometer; glucometer) was reported to be available at 100% of facilities, while this was not the case in the rest of the country. However this study [3] did not assess the availability of IEC materials on NCDs and related risk factors; or barriers to undertaking such health promotion.

Numerous studies have reported that health professionals cite patient non-compliance to lifestyle modification as a barrier that prevents them from providing health promotion on lifestyle modification [2-9]. Paradoxically, the literature provides indisputable evidence that patients view health professionals as reliable and valuable sources of information [10-15] and that patients are more likely to attempt to change their lifestyle in response to advice they receive from health professionals [16-23]. There are many factors that affect patients’ perspectives on health education. These include the scarcity of continuity of care [24,25], receiving conflicting health education messages [14,24], access to the facility in terms of transport [24], accessibility of information in terms of language [24,26] and communication with health professionals as well as cultural beliefs [24,27-31].

In the Western Cape Province of South Africa a large percentage of the population are of mixed ancestry, being of Euro-Afr-Malay origin. The prevalence of NCDs, such as diabetes and heart disease are highly prevalent in this minority population [32], many of whom do not have medical health insurance and have to make use of state health facilities. Another minority group catered for mainly by state health services are the Xhosa-speaking African population. Many of these people have migrated to Cape Town from the Eastern Cape Province. Hypertension and stroke are major causes of mortality in this population [32]. There is however a paucity of data on how minority patients who have a chronic NCD are treated at such facilities. Additionally it is not clear whether these patients receive lifestyle modification education.

The aim of this study was therefore to conduct an audit of the availability and efficacy of equipment as well as health promotion methods and materials currently used by health professionals for the management of patients with NCDs at PHC facilities in Cape Town. Additionally, the study documented barriers preventing the optimal utilisation of health promotion by patients and health professionals.

## Methods

### Study design and sample population

Quantitative and qualitative methods were employed in this 3-phased cross-sectional, descriptive, multi-centre study. The study was conducted in PHC facilities across 11 districts within Cape Town. The study population included health professionals (doctors, nurses, health educators) and patients (mainly of African and mixed ancestral descent), rendering/receiving treatment for one or more NCDs or risk factors at PHC facilities in Cape Town.

### Sampling

Phase 1 was an audit of the equipment and health promotion materials and methods available at PHC facilities with specific reference to diagnosis of NCDs or risk factors. Stratified random sampling, based on geographical location, namely 11 metropolitan health districts, was used to identify a representative sample of PHC facilities (N = 30). Key informants at each facility were purposively selected, to participate in the execution of the audit. Facility managers were required to identify key informants having knowledge on the availability and efficacy of equipment and health education materials.

Phase 2 was a survey of patients attending services for NCDs and/or related risk factors; also health services available and patient preferences for health promotion. Based on the 2003 monthly statistics provided by the Provincial Government of the Western Cape (PGWC), the 30 PHC facilities service an estimated 16 224 and 9 817 hypertensive and diabetic patients respectively. A required sample size of 600 patients was calculated based on a 95% confidence interval and a confidence limit of 0.04. This sample was equally distributed across the 30 facilities, such that 20 (10 diabetic and 10 hypertensive) patients were required at each facility. Diabetics and hypertensive patients were selected since these two conditions are the most commonly found in NCD patients at Cape Town PHC facilities [32].

Phase 3 included a sub-sample of five PHC facilities which were purposively selected based on their location, size, ethnic profile of patients in attendance and the predominant languages spoken at the facility. At each facility where possible, qualitative interviews were conducted with one health professional from three disciplines, including doctors, nurses and health educators. Dietitians/nutritionists were not included in the sample since they are not available at all facilities and secondly the study was to evaluate health professionals who mainly see NCD patients, namely, doctors, nurses and health educators.

#### Data collection

Phase 1: The presence and availability of equipment and health promotion materials were measured by direct observation using a checklist, while the perceived effectiveness of the available health promotion methods and materials and the working condition of equipment were determined by interviews with key informants at each facility. The checklist comprised a list of equipment and materials required to be available at each facility as provided by the facility resource centres.

Phase 2: Each patient included in the sample completed a simple basic questionnaire on their lifestyle habits and NCD risk factors. Furthermore, a semi-structured questionnaire comprising open-ended questions was developed in the local languages (Afrikaans and Xhosa) to identify the PHC services that NCD patients received at PHC facilities and their preferences regarding health promotion materials and methods. Four trained fieldworkers administered the questionnaires to NCD patients who were randomly selected by identifying a primary patient who served as the first interviewee and then selecting every 5th patient in the queue in the waiting room. Patients who declined were replaced by those who were willing to take part.

Phase 3: An interview schedule was developed to explore health professionals’ feelings and the conditions within PHC facilities that facilitate or impede the provision of lifestyle modification education and counselling to NCD patients. Individual semi-structured interviews were conducted with selected health professionals and captured using a digital recorder.

#### Data analysis

Phases 1 and 2 completed questionnaires were coded and computerised. Data were cleaned and analysed using the SAS programme (version 8)(SAS Institute, Cary, NC, USA). Phase 3 interviews were transcribed verbatim. Data were transferred to the Alas ti software programme and analysed using a thematic content analysis approach. The transcripts were reviewed and the data was coded according to predetermined themes and categories.

#### Ethics

Ethical clearance was obtained from the Ethics Committee at the University of Cape Town (REC REF: 170/2004). Permission to conduct the study was obtained from the Community Health Services Organisation in the Western Cape and informed consent was provided by each participant.

## Results and discussion

### Profile of patients interviewed

The projected sample size required was 600 patients while the realised sample was 580 patients; 29% (N = 171) were male and 71% (N = 409) were female, with a mean age of 55 ± 10 years (ranging from 21 to 84 years). Overall 55% and 81% reported receiving treatment for diabetes and hypertension respectively. Overall 49% of diabetic and 73% of hypertensive patients had received treatment for their chronic conditions at their current PHC facilities for more than a year. The majority (78%; N = 452) reported eating at least 3 meals per day, more than half (56%; N = 325) of whom include snacking as part of their daily meal pattern. A large proportion (57%) further indicated that they perceived that they eat more than they require. The majority of patients (65%) reported that overall they were moderately active; while 29% reported that they were inactive. Additionally, 81% reported that they were inactive when they were not at work (weekends and evenings), while only 18% reported being moderately active.

Twenty-three percent of patients indicated that they smoke cigarettes, 71% that they did not smoke and 6% indicated that they used to smoke but had quit smoking. Of the 23% (n = 133) that indicated they smoked, 69% reported smoking 1 - 9 cigarettes per day, while 25% reported smoking 10 - 19 per day and 5% reported smoking 20 or more per day. Fourteen percent of patients reported that they consumed alcohol, 78% reported that they do not consume alcohol while 8% reported that they had quit drinking alcohol. Of the 14% (n = 81) that reported drinking alcohol, 87.5% reported that they did not drink during the week, 7.5% reported having 2 – 3 drinks (standard alcohol exchanges)[33] per week while 5% reported having 1 – 2 drinks per day. Twelve percent of these patients reported that they did not consume alcohol on weekends, while 21%, 35% and 32% reported that they have 1 - 2 drinks per day, 2-3 drinks per day and 3 or more drinks per day respectively.

### Availability and efficacy of equipment at PHC facilities required to deal with NCDs

The majority of PHC facilities reported that they had access to at least one adult scale, glucometer, haemoglobin meter, baumanometer, visual acuity chart and ophthalmoscope in a good working condition, as reported earlier in the national study by Reagon *et al.*[3](Table 1). Equipment that was not available at all facilities included height measures, BMI charts, tape measures, large blood pressure cuffs, televisions and video machines. However, at facilities where equipment was available it was often reported to be in short supply. Furthermore, some facilities reported inadequate access to consumables such as glucose strips and batteries for glucometers. Most of the available equipment was reported to be in a good working condition. However, a large proportion of facilities indicated that equipment such as scales, height measures and visual acuity charts were in need of maintenance, repair or replacement.

**Table 1:** Summary of equipment available at health facilities (n = 30)

**Equipment**	**Available at all facilities**	**No. (%) of facilities with 0 items**	**No. (%) of facilities with 1 item**	**No. (%) of facilities with 2 items**	**No. (%) of facilities with 3 items**	**No. (%) of facilities with 4 items**	**No. (%) of facilities with 5 + items**	**Max no. of items at facility**
Adult scale	Yes		11 (37)	11 (37)	4 (13)	2 (7)	2 (7)	6
Height measures	No	4 (13)	13 (43)	9 (30)	3 (10)	0 (0)	1 (3)	8
BMI charts	No	13 (43)	11 (37)	2 (7)	3 (10)	0 (0)	1 (3)	15
Tape measures	No	16 (53)	8 (27)	2 (7)	3 (10)	1 (3)	0 (0)	4
Hb meters	Yes		9 (30)	14 (47)	3 (10)	3 (10)	1 (3)	6
Glucometers	Yes		16 (53)	8 (27)	2 (7)	2 (7)	2 (7)	7
Baumonometers	Yes		1 (3)	0 (0)	3 (10)	2 (7)	24 (80)	17
BP cuffs	Yes		1 (3)	0 (0)	3 (10)	2 (7)	24 (80)	17
Large BP cuffs	No	5 (17)	8 (27)	8 (27)	6 (17)	3 (10)	0 (0)	4
Visual acuity charts	Yes		9 (30)	11 (37)	3 (10)	5 (17)	2 (7)	6
Opthalmoscopes	Yes		0 (0)	2 (7)	7 (23)	3 (10)	18 (60)	15
Television	No	3 (10)	21 (70)	3 (10)	2 (7)	0 (0)	1 (3)	5
Video machines	No	4 (13)	21 (70)	4 (13)	1 (3)	0 (0)	0 (0)	3

The reality at PHC facilities is that there is a scarcity of equipment within facilities. This often results in overuse and abuse of available equipment which ultimately leads to the constant repair and maintenance of equipment. Ideally each health professional should be issued with their own equipment for which they are responsible. Furthermore a schedule for maintenance of equipment should be established and implemented.

### Screening services rendered at PHC facilities

Blood pressure measurement (97.6%) was the most common diagnostic test practiced, followed by weight measurement (88.3%), urine testing (85.7%) and blood glucose testing (80.9%). However, less than 50% of patients reported that their height had been measured. This implies that health professionals at these facilities did not calculate or interpret patients’ body mass index (BMI) at the time of the study. About a quarter (26.4%) of patients reported having an ECG performed on them. Although 55% of patients reported that they had diabetes, at the time the survey was conducted, retinal screening and diabetic foot screening at these facilities was reported by less than 20% of patients. Similar results were reported for cholesterol tests (17.9%), reflex tests (16.6%), chest x-rays (12.1%) and kidney function tests (11.4%). Only 7.4% of patients reported that their waist circumference had been measured, while 15.9% of patients reported that they had been referred to a tertiary hospital. These results indicate that the patients’ examinations were not always comprehensive. This may be due to the fact that PHC in SA is still based on the medical model which is more appropriate for treating patients with acute conditions while it does not cater adequately for patients with chronic conditions despite the fact that chronic patients comprise at least 50% of attendees at PHC facilities [3]. Health professionals are inclined to focus on the more urgent and immediate needs of acute care patients, even at the expense of doing less comprehensive screening tests on chronic patients.

#### Health promotion methods and materials employed at PHC facilities

**Figure 1:**
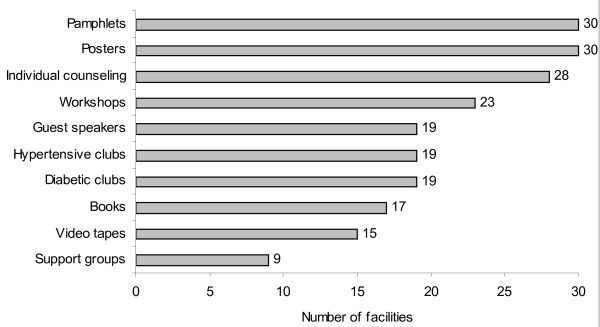
Health promotion methods available at PHC facilities (n = 30).

The range of health promotion methods available at PHC facilities included educational posters, pamphlets (3-4 pages), books/booklets, use of clubs or support groups, individual counselling sessions, group counselling sessions and employment of guest speakers. These are summarised in descending order of availability in Figure 1. The three most frequently used methods were educational posters (n = 30), pamphlets (n = 30) and individual counselling (n = 28), while the availability of support groups were the least used method.

Table 2 provides a summary of the awareness and availability of health promotion materials (charts, guidelines, videotapes, pamphlets and posters) at PHC facilities. The most disturbing finding was that the Department of Health National Guidelines for Prevention and Management of NCDs were either not available or else only available at one out of the 30 facilities. These guidelines had been developed by experts in order to assist health care staff in dealing with NCD management in a standardised manner. Pamphlets and posters for patients also were not optimally utilized, with a large number of facilities not knowing about their existence or having them available at the facility. Although the majority of facilities have televisions and video machines, a mere 5 facilities reported using videotapes as a method of health promotion. This could explain that awareness of NCD videos available at the PGWC resource centres was minimal at all facilities.

**Table 2:** Awareness and availability of health promotion materials on NCD prevention at 30 primary health care facilities in Cape Town

	No. of facilities aware of item*	**No. of facilities which have the item**
i) Wall charts with standard guidelines for diagnosis & treatment		
- Hypertension	24	26
- Diabetes	13	13
ii) Booklets		
- Diabetes	26	16
- High blood pressure	13	6
iii) Department of Health National guidelines on:		
- Control & management of type 2 diabetes	18	1
- Stroke & transient ischemic attack management	4	1
- Primary prevention of chronic diseases of lifestyle	2	0
- Osteoporosis	1	0
- Prevention & management of overweight & obesity	0	0
- Guidelines for healthy eating	7	1
iv) Pamphlets on:		
- Diabetes (9 different ones available)	0-19	0-17
- Hypertension (4 different ones available)	13- 20	8-16
- Alcohol (1 available)	12	9
- Nutrition (3 different ones available)	11-14	8-11
- Heart disease/cholesterol (4 different ones available)	10- 14	8-9
- Smoking (2 different ones available)	7- 14	6-10
-Physical activity (1 available)	10	5
- Obesity (2 different ones available)	11-17	8-16
v) Posters		
- Diabetes (9 different ones available)	12-26	7-20
- Hypertension (4 different ones available)	6-17	2-11
- Alcohol (3 different ones available)	11	3-5
- Nutrition (3 different ones available)	11-14	8-11
- Smoking (2 different ones available)	1	1
vi) Videotapes		
- Chronic diseases	1-6	5**
- Obesity & physical activity	0-5	5
- Heart disease and stroke	0-4	5
- Nutrition	2-13	5
- Smoking	1-2	5

*The range differed between minimum and maximum number of different items.

**Only 5 facilities showed videos.

### Patient preferences for health promotion materials and methods

Ninety percent of patients (N = 522) reported that they wanted information, while the remaining 10% did not. The reasons provided by the latter (N = 58) included not being interested in receiving information (21%), being informed already (31%), being able to manage their condition well (12%) and a lack of time to obtain information (10%). Some patients also reported that they felt that it was too much for them to comprehend stating that “*there's so much information about everything sometimes it’s too much for people to take in.”*

Patients were presented with a list of seven possible health promotion methods and materials, from which they were requested to select their four favourite methods and to rate them in order of preference. All methods were selected, albeit to varying degrees (Table 3). Individual counselling emerged as the preferred method of 71% of patients, of which 56% rated it as their first choice. Watching a video was chosen by 69% of patients, however, only 22% rated it as their first choice. Of the 64% of patients that selected chronic clubs/support groups, only a third rated this as their first choice, with a large proportion (40%) rating it as their second choice. Even though 58% of patients chose booklets as their preferred method of choice, almost half (42%) these patients rated this as their fourth (last) choice. Pamphlets, posters and workshops/group counselling sessions were the least preferred methods with only 9%, 13% and 11% of patients choosing these as their first choice respectively.

**Table 3:** Patients’ ratings of preferred health promotion methods and materials (%)

**Health education method/materials**	**Overall choice Percentage (N)**	**1st choice %**	**2nd choice %**	**3rd choice %**	**4th choice %**
Individual counselling	71 (414)	56	17	15	12
Watching a video	69 (398)	22	26	23	30
Club or support group	64 (371)	31	40	18	11
Booklets	58 (336)	19	16	23	42
Pamphlets	52 (303)	9	19	41	32
Posters	43 (250)	13	34	25	28
Workshop/group counselling	39 (227)	11	26	40	24

Some of the reasons provided for selecting individual counselling included *“being able to ask questions freely, without feeling intimidated”* and *“receiving information that is specific to my individual consultation and examination*”. Those who selected chronic clubs, support groups or group counselling sessions stated that *“being with people like me would motivate me because I’m not alone in my condition”*, furthermore it provided them with the opportunity to *“communicate with patients who are in need of support and encouragement”* because *“everybody who goes there has the same problems and you can share your experiences”*.

Reasons for selecting videotapes included the fact that they are visually demonstrative such that *“because you can see it, you can understand it better”*. Furthermore it would be convenient to watch a video at the facility since *“people sit here the whole day, they wait very long and a video could relieve their boredom and make them less frustrated”*. Patients further stated that it would be an *“ideal way of educating large numbers of people at the same time”* and in doing so it could *“reduce the amount of time doctors and nurses need to spend on health education”*.

The most common reason provided for selecting booklets or pamphlets was the opportunity to *“take information away from the facility and read it in my own time”*. It also allowed them to *“share the information with people outside the facility”*. Patients who were illiterate stated that *“my children can read it to me so that I can understand it better”.* The few who selected posters, did so because it is easily accessible *“you see them all over in the hospital”* and it’s effective because *“they are short and to the point, people will remember a short message rather than a long story”*. In addition they are illustrative thus *“you can understand what is going on just by looking at the pictures, you don’t even have to read the words”*.

#### Preferred provider of health promotion

Approximately 30% of patients had no preference regarding which health professional provided education in three settings, namely, individual counselling, chronic clubs/support groups and workshops or group counselling sessions (Table 4). However, in an individual counselling setting 44.7% of patients reported that they would prefer to be counselled by a doctor, followed by a nurse (16.9%), the health educator (8.8%) and least a nutrition advisor (lay nutrition educator)(4.8%). In a club or support group setting, doctors (24.3%) and nurses (26.7%) were the preferred choices while health educators (11.2%) and nutrition advisors (6.9%) were the least likely choices. Patient choices at workshops/group sessions closely resembled those in a club or support group setting with 24.5%, 21.2%, 14.7% and 5.7% choosing doctors, nurses, health educators and nutrition advisors respectively.

**Table 4:** Patients’ preferences regarding which health professional should provide information

**Preferred health professional**	**Percentage of patients (N = 580)**		
	**Individual counselling**	**Chronic Club or Support group**	**Workshops**
Doctor	44.7	24.3	24.5
Nurse	16.9	26.7	21.2
Health educator	8.8	11.2	14.7
Nutrition advisor	4.8	6.9	5.7
Anyone of the above	24.8	30.9	34.0

One of the leading factors that compromise service delivery is the inadequacy of the staff component within PHC facilities. The present study echoes this need as the shortage of staff was identified as a barrier that compromised service delivery. The staff shortages can be largely attributed to the “brain drain” of health professionals from the public to the private sector and abroad [34]. Currently, the nursing staff at PHC facilities in SA work on a rotation basis, whereby they work in different departments and with different diseases. Although this system can be successfully applied within an acute care system a chronic care system requires a more specialised training in chronic care management and dedicated chronic care teams are required.

### Health professional perspectives

The majority of health professionals reported that they perceived lifestyle modification to have a positive role in the management of NCD patients; “*cannot treat a patient with drugs alone…lifestyle modification is extremely important… more than 50% of what you supposed to do… most of the patients do well with lifestyle modification without medication” Doctor - Gugulethu and “…I will still say it’s the cornerstone of trying to manage any chronic disease… whether it’s diet, exercise, not smoking, not drinking… that is vital” Doctor - Mitchells Plain.*

In addition, health professionals across all cadres indicated that they were responsible for patient education and empowerment. They were more likely to provide individual counselling to newly diagnosed patients and those whose conditions were poorly controlled. The content of these sessions mainly centred on information regarding disease conditions, medication as well as lifestyle modification associated with the disease. For those patients who were well controlled, they were more likely to *“build support groups to make people understand their disease and take control of it” Doctor - Gugulethu* – such that they do not rely solely on health professionals but play an active part in managing their chronic conditions.

The findings of the present study indicates that it is easy for individual staff members to lose sight of the importance of their role in the management of chronic patients, however, when they attend workshops or group meetings they are reminded of the importance of their individual roles and as such become motivated to provide improved services to patients. Reinforcing the roles of individual health professionals in the management of chronic patients thus significantly impact on the quality of care that health professionals provide. A study in Australia with aborigines [35] echoed this need as they reported that regular meetings amongst chronic care teams were required to revise and reinforce the roles of all the team members.

#### Access to, and effectiveness, of health promotion materials and methods

In order for health professionals to provide effective education and counselling services to NCD patients they require adequate access to effective health promotion materials and methods.

The majority of health professionals experienced difficulty in accessing health promotion materials. The main difficulties were a scarcity of materials at the provincial resource centre; limited availability of materials in local languages; and a lack of transport to collect materials. The scarcity of materials compelled health professionals to do selective distribution of materials to patients and health professionals often resorted to photocopying old materials, resulting in illegible materials. Health professionals thus tended to scout for materials from alternate sources such as private health facilities, pharmaceutical companies, and non-government organisations.

One of the issues which arose in the current study was that of access to and efficiency of health promotion materials and methods. The one outstanding finding regarding health promotion materials was that there is an abundance of materials that have been designed by numerous sources, but that the awareness and presence of these materials was not optimal. This includes the National Guidelines for the management of chronic diseases. Conversely, the presence of materials developed by pharmaceutical companies is far more common since they are delivered directly to the facilities by the companies’ representatives. Since the Department of Health has invested time and money in developing education materials and management guidelines they should invest further by improving the distribution of these materials to facilities by ensuring efficient delivery of materials.

With regard to the perceived effectiveness of health education materials, health professionals tended to agree that posters and pamphlets are practical and easily accessible due to their high visibility within the facility. They further stated that they are easy to read and understand since they are usually illustrated, making it easier for patients to remember the information. The greatest shortcoming regarding these methods was the fact that a large proportion of patients attending these facilities are illiterate and may have poor eyesight, thus rendering these methods completely ineffective. Furthermore, posters and pamphlets are often stolen or damaged.

When reviewing the effectiveness of health education methods, individual counselling was the method that received the most positive reviews, with health professionals stating that this method may be the largest contributory factor to patient compliance. Group counselling sessions were perceived to be effective; however, they do not afford patients the opportunity to ask personal questions since they are structured so that staff are usually outnumbered by patients in these sessions and as a result they may not always be able to retain the attention of all the patients attending these sessions. Thus if *“the ratio of the staff to the patient if it is reduced the patient will tend to listen and look at the nurses better, they will be more serious than if they are talking to somebody in a group where some people will be making noise and not listen ..*” *Doctor - Gugulethu*

Health professionals reported that support groups provide an ideal environment for interaction between fellow NCD patients and allows them to encourage and support each other. This empowers patients to take responsibility for their own health and educate other people within their communities about their conditions.

### Factors that motivate health professionals to provide education

Various factors motivate health professionals to provide education to NCD patients. Some of these include professional responsibility, “*doctors are clinicians they diagnose disease, tell the person then manage it so its part of the management of the patient” Doctor – Gugulethu;* caring for patients, “*you care about people… you want them to make changes to their lifestyle” Doctor - Lady Michaelis* and having firsthand experience of living with NCD, “*mostly what motivates me is also that I'm also diabetic, hypertensive and asthmatic…I know about the diseases because I'm also affected by them” Nurse - Gugulethu*

Patient adherence and positive results further motivate them, *“when we get result and gratitude from patients in the chronic clubs… you excited to see that the effort you put in is helping the patient” Nurse – Ruyterwacht.* Conversely the mortality and morbidity associated with NCDs also motivates them to provide education and counselling to patients as a means of reducing the associated burden placed on the health services, *“I've been to many hospitals, I’ve seen amputations and people going blind… our hospitals are not equipped for stroke patients… we should educate patients, because prevention is better than cure” Nurse- Mitchells Plain.* In the same way, providing education and counselling to patients would enable them to manage their conditions better and thus experience fewer complications, thereby reducing the doctors’ workload.

### Barriers preventing effective education and counselling

Health professionals identified numerous barriers to education and counselling. These can be summarised as a lack of resources, including time, space and equipment; staff-related barriers such as staff shortage and staff turnover and patient-related barriers (patient load and patient non-compliance) among others. Table 5 compares the identified barriers across the three health disciplines and clearly illustrates that doctors reported experiencing far more barriers, especially those related to staff, services and language, than both the nurses and health educators.

**Table 5:** Barriers to health promotion by different professions (n = 14)

**Category**		**Doctors**	**Nurses**	**Health educators**
Resources	Time	8	4	3
	Space	4	6	1
	Equipment	2	2	3
	Services	4	0	0
Staff	Staff shortage	4	3	2
	Other responsibilities	1	2	3
	Attitude	3	0	0
	Lack of knowledge	1	0	0
	Fatigue (repetition)	1	0	0
	Lack of referral to health professionals	0	0	1
	Rotations	1	0	0
	Turnover	1	0	0
Patients	Load	2	4	0
	Non-compliance	2	3	0
	Attitude	2	0	1
	Other health problems	3	0	0
Other barriers	Language	3	0	0
	Crime	0	1	1
	Implementation of chronic clubs	1	0	1
		44	27	16

Lack of time appeared to be the most common barrier cited, however, there are numerous underlying factors that contribute to this. The staff : patient ratio is at the forefront of the factors. Doctors reported that *“very often nurses are short staffed… they have to do other things, they don't have time to do health education…” Doctor - Lady Michaelis*, while nurses reported that “*there's a lot of patients up to 400 - 500 a day… you don't have time to counsel them” Nurse – Rusthof*; and health educators stated that *“I would like to reach everybody but it's impossible because I'm a one person department” Nurse- Lady Michaelis*, as a result doctors reported that *“you end up spending only 5 minutes or even less with each patient which is not enough to educate them on what he needs to do” Doctor - Gugulethu*

Language barriers also contributed to the lack of time. If available, interpreters are utilized to bridge the language barriers between staff and patients. However, one of the doctors stated that this often wastes time because the interpreter may not always convey the exact message that the doctor or the patient is trying to convey.

One of the doctors mentioned that some staff requires further training in order to provide efficient education and counselling to NCD patients, while others state that *“…as a caregiver you get tired of saying the same thing over and over and over again so maybe the first ten patients would get a lot of education and the ones that you see in the afternoon when you’re tired not as much” Doctor - Rusthof*

With respect to patients, non- adherence to lifestyle modification principles presents a large barrier to education and counselling. They state that it is very frustrating to spend time counselling patients in vain. One of the health professionals mentioned that although the patients listen to the advice provided while they are at the facility, when they go home they return to their routine lifestyles. She added that a contributory factor to patient non-adherence is the fact that *“here things are cheap that's why some cannot control their diseases… everything they not supposed to eat is cheaper here” Nurse – Gugulethu.*

### Suggestions to facilitate health education and counselling

Firstly, health professionals suggested increasing the overall size of the facility in order to provide more space, *“if you really want health promotion to work 120% give us space… when you do talks you must stand in the passage… if you've got a separate room, you will get better response from the patient” Nurse- Mitchells Plain*

Suggestions related to staff included increasing the staff complement; *“more time with individual patients… that means we need more staff so that we don’t have to say we can only spend so much time with you before we need to call the next person” Nurse- Lady Michaelis*, reducing staff rotations, *“a person in chronic care should be allowed to stay there for at least one year because rotating staff in the club makes things chaotic… people keep changing ideas and things never get done because it will never be the first priority” Doctor - Gugulethu* and providing dedicated NCD staff, *“people want to see one doctor, they want to see the same nurses…so if there can be dedicated nurses for the chronic club” Nurse - Mitchells Plain*

Furthermore, health professionals requested access to continuous education in the form of workshops, *“you tend to forget sometimes so to go to workshops where they work from the basics of chronic care and then discuss new developments” Doctor - Lady Michaelis.* Workshops could also boost staff morale and highlight each health professional’s role in the management of a NCD patient, *“previously the nursing staff they didn't see their role in the management of the patient. But once they went to the workshop they got the overall picture and then they became more motivated” Doctor - Mitchells Plain*

Suggestions regarding health education material mainly focussed on improved access to materials, especially culturally and language appropriate materials. With respect to health education methods, health professionals requested improved access to audiovisual equipment and audiovisual aids.

## Conclusion

In conclusion, the majority of patients attending PHC facilities want to receive lifestyle modification education. There is not however, one specific method that can act as a gold standard for providing education to NCD patients. Patients’ preferences regarding health education methods differ, and they are more likely to be susceptible to methods that do not involve reading. Health education materials such as posters, pamphlets and booklets should be used to supplement information received during a counselling or support group sessions. Taking cognisance of patients’ preferences for health promotion materials and methods by improving access to health videos and support groups/chronic clubs will result in increased patient compliance to health promotion messages and thus improve education and counselling services provided at PHC facilities.

## Competing interests

No conflict of interest is declared and no industry funding was utilized in this research which was funded by the South African Medical Research Council.

## Authors’ contribution

W Parker was the principle researcher and author. NS and NL were co-authors and co-investigators. CJL was responsible for the sampling and statistical analyses. All authors read and approved the final manuscript.

## Pre-publication history

The pre-publication history for this paper can be accessed here:

http://www.biomedcentral.com/1471-2458/12/503/prepub

## References

[B1] WHO: Global Strategy on Diet 2004 Physical Activity and Health, Geneva Accessed November 18th 2009, http://www.who.int/dietphysicalactivity.org

[B2] Chopra M Steyn K Lambert EV Western Cape burden of disease reduction project::vol 6 of 7. Decreasing the burden of cardiovascular disease 2007 Department of Health, Pretoria

[B3] Goodman GR Zwarensyein MF Roninson II Levit NS Staff knowledge, attitudes and practices in public sector primary care of diabetes in Cape Town S Afr Med J 1997 87 3 305 309 9137343

[B4] Moore H Adamson AJ Gill T Waine C Nutrition and the health care agenda Fam Pract 2000 17 2 197 202 10.1093/fampra/17.2.197 10758086

[B5] Jallinoja P Pilvikki A Kuronen R Nissinen A Talja M Uutela A Patja K The dilemma of patient responsibility for lifestyle change: Perceptions among primary care physicians and nurses Scand J Prim Health Care 2007 25 244 249 10.1080/02813430701691778 17934984 PMC3379767

[B6] Aira M Kauhanen J Larivaara P Rautio P Factors influencing inquiry about patients’ alcohol consumption by primary health care physicians: qualitative semi-structured interview study Fam Pract 2003 20 270 275 10.1093/fampra/cmg307 12738695

[B7] Wens J Vernweire E van Royen P Sabbe B Denekens J GP’s perspectives of type 2 diabetes patients’ adherence to treatment: A qualitative analysis of barriers and solutions BMC Fam Pract 2005 6 20 10.1186/1471-2296-6-20 15890071 PMC1156882

[B8] Alberti H Boudriga N Nabli M Primary care management of diabetes in a low/middle income country: A multi-method, qualitative study of barriers and facilitators to care BMC Fam Pract 2007 8 63 10.1186/1471-2296-8-63 17996084 PMC2186326

[B9] Douglas F Torrance N van Teijlingen E Meloni S Kerr A Primary care staff's views and experiences related to routinely advising patients about physical activity, A questionnaire survey BMC Public Health 2006 6 138 10.1186/1471-2458-6-138 16719900 PMC1523207

[B10] Kolasa KM “Images” of nutrition in medical education and primary care Am J Clin Nutr 2001 73 1006 11382652 10.1093/ajcn/73.6.1006

[B11] van Dillen SME Hiddink GJ Koelen MA de Graaf C van Woerkum CMJ Understanding nutrition communication between health professionals and consumers: development of a model for nutrition awareness based on qualitative consumer research Am J Clin Nutr 2003 77 suppl 1065S 1072S 12663320 10.1093/ajcn/77.4.1065S

[B12] Hiddink GJ Hausvast JG van Woerkum CM Fieren CJ van’t Hof MA Consumer’s expectations about nutrition guidance: the importance of primary care physicians Am J Clin Nutr 1997 65 Suppl 1974S 9S 9174506 10.1093/ajcn/65.6.1974S

[B13] Serra-Majem LL Calvo JR Male ML Ribas L Lainez P Population attitudes towards changing dietary habits and reliance on general practitioners in Spain Eur J Clin Nutr 1999 53 Suppl 2 S58 S61 10406439 10.1038/sj.ejcn.1600804

[B14] Buttriss JL Food and nutrition: Attitudes, beliefs and knowledge in the United Kingdom Am J Clin Nutr 1997 65 (6) Suppl 1985S 1995S 9174508 10.1093/ajcn/65.6.1985S

[B15] Pelto GH Santos I Goncalves H Victora C Martines J Habicht JP Nutrition counselling training changes physician behaviour and improves caregiver knowledge acquisition J Nutr 2004 134 357 362 14747672 10.1093/jn/134.2.357

[B16] Westa R McNeillb A Rawe M Smoking cessation guidelines for health professionals: an update Thorax 2000 55 987 999 10.1136/thorax.55.12.987 11083883 PMC1745657

[B17] Thun M Improving the treatment of tobacco dependence Br Med J 2000 321 311 10.1136/bmj.321.7257.311 10926568 PMC1118311

[B18] Harris SS Caspersen CJ DeFriese GH Estes EH Physical activity counselling for healthy adults as a primary preventive intervention in the clinical setting JAMA 1989 261 3590 2657126

[B19] Allen SS Harris IB Kofron PM Anderson DC Bland CJ Dennis T Satran L Miller WJ A comparison of knowledge of medical students and practicing primary care physicians about cardiovascular risk assessment and intervention Prev Med 1992 21 4 436 48 10.1016/0091-7435(92)90052-J 1409486

[B20] Lancaster T Stead LF Physician advice for smoking cessation The Cochrane Database of Systematic Reviews 2004 4 10.1002/14651858.CD000165.pub3 18425860

[B21] Peiss B Kurleto B Rubenfire M Physicians and nurses can be effective educators in coronary risk reduction Gen Intern Med 1995 10 2 77 81 10.1007/BF02600231 7730943

[B22] Kreuter MW Chheda SG Bull FC How does physician advice influence patient behaviour? Evidence for a priming effect Arch Fam Med 2000 9 5 426 433 10.1001/archfami.9.5.426 10810947

[B23] Egede LE Lifestyle Modification to improve blood pressure control in individuals with diabetes. Is physician advice effective? Diabetes Care 2003 26 3 602 607 10.2337/diacare.26.3.602 12610008

[B24] Tripp-Reimar T Choi E Skemp Kelley L Enslein JC Cultural barriers to care: inverting the problem Diabetes Spectrum 2001 14 13 22 10.2337/diaspect.14.1.13

[B25] Berry LL Parish JT Janakiraman R Ogburn-Russell L Couchman GR Rayburn WL Grisel J Patients’ commitment to their primary physician and why it matters Ann Fam Med 2008 6 1 6 13 10.1370/afm.757 18195309 PMC2203391

[B26] Lukoschek P Fazzari M Marantz P Patient and physician factors predict patients’ comprehension of health information Patient Education and Counselling 2003 50 201 210 10.1016/S0738-3991(02)00128-3 12781935

[B27] Boult L Boult C Underuse of physician services by older Asian-americans J Am Geriatr Soc 1995 43 408 411 7706632 10.1111/j.1532-5415.1995.tb05816.x

[B28] Tom-Orme L Joe JR, Young RS Traditional beliefs and attitudes about diabetes among Navajos and Utes Diabets as a Disease of Civilization: The Impact of Culture Change on Indigenous Peoples 1993 Mouton de Gruyter, New York 272 291

[B29] Wong C Llorens LA, Hikoyeda N, Yeo G Models of prevention or management programmes targeted to specific ethnic elders: Chinatown Health Centre Diabetes Among Elders: Ethnic Considerations 1992 Stanford Geriatric Education Centre, Stanford 70 72

[B30] Puoane T Fourie JM Shapiro M Rosling L Tshaka NC Oelefse A ‘Big is beautiful’ – an exploration with urban black community health workers in a South African township S Afr J Clin Nutr 2005 18 1 6 15

[B31] Nthangeni G Steyn NP ALbetrs M Steyn K Levitt NS Laubscher R Bourne L Dick J Temple N Dietary intake and barriers to dietary compliance in black type 2 diabetic patients attending primary health care services Public Health Nutr 2002 5 2 329 338 12020385 10.1079/PHN2002256

[B32] Bradshaw D Groenewald P Laubscher R Nannan N Nojilana B Norman R Pieterse D Schneider M Bourne DE Timaeus IM Dorrington R Johnson L Initial burden of disease estimates for South Africa, 2000 S Afr Med J 2003 93 9 682 686 14635557

[B33] Shafer S Diabetes type 2--complete food management program 2001 American Diabetes Association, New York Alcohol. Retrieved April 29, 2004, from: http://www.diabetes.org/utils/printthispage.jsp?PageID=TYPE1DIABETES3_232989

[B34] SAInfo:Health care in South Africa , Available at http://www.southafrica.info/about/health/health.htm (21/4/2012)

[B35] Bailie R Si D Dowden M O’Donoghue L Connors C Robinson G Cunningham J Weeramanthri T Improving organisational systems for diabetes care in Australian indigenous communities BMC Health Services Research 2007 7 67 10.1186/1472-6963-7-67 17480239 PMC1876220

